# Identification of Risk Factors Associated with Treatment for BRD in Beef Calves Within the First 60 Days After Arrival at Fattening Operations in Northwestern Italy Beef Calves

**DOI:** 10.3390/vetsci12090898

**Published:** 2025-09-16

**Authors:** Isabella Nicola, Giuliano Borriello, Edoardo Ramacciotti, Giovanni Gallina, Maurizio Beltramo, Claudio Bellino

**Affiliations:** 1 Faculté de Médecine Vétérinaire, Université de Montréal, Saint-Hyacinthe, QC J2S 2M2, Canada; isabella.nicola@umontreal.ca; 2 Department of Veterinary Sciences, University of Turin, Largo Paolo Braccini 2, 10095 Grugliasco, Italy; 3 Independent Researcher, 14020 Pieia D'Asti, Italy; 4 Independent Researcher, 15034 Cella Monte, Italy; 5 Independent Researcher, 12032 Barge, Italy

**Keywords:** antimicrobial use, respiratory disease, risk factor, welfare

## Abstract

Bovine respiratory disease (BRD) impacts beef cattle health and farming systems. To better understand the BRD predisposing factors, a study was conducted in northwest Italy on a population of 26 batches (760 animals) of beef cattle imported from France. Of these, 173 underwent physical examination for clinical signs of BRD (rectal temperature > 39.5 °C, respiratory rate > 36 bpm, cough, nasal or ocular discharge) and blood sampling to detect antibodies against bovine viral respiratory infectious agents (e.g., parainfluenza 3, bovine herpesvirus type 1), haptoglobin and reactive oxygen metabolites concentrations, on arrival. Data on BRD treatments performed within 60 days of arrival were extracted from farm registers. The two most frequent cattle breeds were Blonde d’Aquitaine (80/173, 46.2%) and Limousine (61/173, 35.3%); the median batch weight was 332 kg (range, 195–470). At least one clinical sign of BRD was noted in 57.2% (99/173) of the animals. Most animals tested positive for BPIV3 (131/173, 75.7%) and BRSV (112/172, 64.7%). Bovine respiratory disease treatment was associated with transport time and average weight on arrival. Moreover, reactive oxygen metabolites levels differed between treated and untreated animals; this difference could help predict the onset of BRD, though further studies are needed to draw conclusions.

## Simple Summary

Bovine respiratory disease (BRD) is among the most important health issues in beef cattle, as it causes illness and economic losses. Several risk factors cause the onset of this disease, and understanding them is an important step for BRD prevention. Therefore, a study was conducted in northwest Italy on 760 beef cattle imported from France. The cattle were grouped into 26 batches. On arrival at the farm, 173 animals were checked for signs of BRD (e.g., coughing, tachypnea) and subjected to blood sampling to detect antibodies against common viruses related to respiratory diseases, and to measure levels of haptoglobin and reactive oxygen metabolites. Data on antibiotic treatment for BRD performed within 60 days of arrival were collected. The most common breeds examined were Blonde d’Aquitaine and Limousine. Over half of the examined animals (57.2%) showed at least one sign of BRD and many tested positive for BPIV3 (75.7%) and BRSV (64.7%). The need for treatment was related to longer transport times and lower weight on arrival. Differences in oxidative stress levels were also found between treated and untreated animals. These differences could help predict BRD risk, although more studies are needed to confirm these findings.

## 1. Introduction

The bovine respiratory disease (BRD) affects beef cattle farming systems and is one of the primary causes of economic loss to the industry. BRD onset is related to diverse stress factors that calves are exposed to [1,2]. For example, abrupt weaning, transport to new facilities, and commingling with other calves especially during the first days after arrival can increase blood levels of cortisol and catecholamine [3,4]. Indeed, such events impair the immune system of young animals, leading to a low immune response and colonization of the lower respiratory tract by pathogenic or opportunistic microorganisms [5]. In addition, slow average daily gain, pharmacological treatment, and higher mortality rates [6] make understanding the risk factors for BRD crucial in its prevention and management.

Identifying risk factors and early indicators for BRD development could help improve the management of different fattening periods and promote more judicious use of antimicrobials in these operations.

Owing to its multifactorial nature, risk factors for the development of BRD have been correlated with animal health and the environment [7]. For instance, weight, sex or breed may influence susceptibility to BRD [4]. Lighter-weight calves may be more susceptible [8,9], while females are reported to be less at risk for morbidity and mortality [10,11]. Furthermore, calves reared in beef fattening operations are often subject to environmental stress factors during the first days on feed, when the incidence of BRD is reportedly highest [11]. Among other factors [4,12,13,14], commingling poses a source of stress and exposes immune-naïve calves to pathogens [8]. Viral infection is recognized as preliminary to bacterial respiratory tract colonization [15]. The most frequently identified viruses in BRD are bovine respiratory syncytial virus (BRSV), bovine parainfluenza 3 virus (BPIV3), bovine alphaherpesvirus type 1 (BoAHV-1), and bovine viral diarrhea virus (BVDV) [15].

Previous studies reported a correlation between seroconversion during the fattening period and higher incidence of BRD. However, animals with confirmed seropositivity prior to arrival were noted to be at lower risk of developing BRD in Australian and Canadian feedlots [16,17]. Moreover, disease onset or a stressful event can lead to an increase in serum acute phase proteins (APP), which are non-specific markers of tissue damage [18]. Their levels increase during the course of BRD, regardless of the severity of clinical signs, and after transport or abrupt weaning. Haptoglobin has been reported to be specific for identifying sick animals because it is less sensitive to stress factors and a better indicator of the degree of inflammation than other acute phase proteins [19,20,21].

Oxidative stress-mediated responses during animal transport [22] occur due to reactive oxygen metabolite production exceeding antioxidant system compensation [23]. Commonly involved in physiological processes, reactive oxygen metabolites possess bactericidal properties but may become harmful at high concentration [23,24]. These highly reactive molecules can react with lipids, proteins, and carbohydrates, thus altering cellular functional or leading to necrosis, with tissue damage, metabolic alteration or impaired immune response [23,24]. In a previous study, elevated oxidative stress following transportation was determined by measuring antioxidant potential and lipid peroxidation products [22]. The study reported an association between higher concentrations of oxidative stress biomarkers and treatment for BRD or mortality [22].

Identifying potential early predictors of BRD can support improved animal management. While haptoglobin has already been tested, albeit with controversial results, to the best of our knowledge, the reactive oxygen metabolite concentration has not been investigated as a predictor for BRD treatment. Therefore, in the present study, we investigated factors predisposing for BRD treatment during the first 60 days of the fattening period and the potential use of haptoglobin and reactive oxygen metabolite levels as predictors of treatment. We hypothesized that some risk factors and potential indicators of subsequent BRD development could be identified, which may help farmers better manage the fattening period.

## 2. Materials and Methods

### 2.1. Farm Selection

This prospective cohort study was carried out in 2015 in collaboration with the Association of Agricultural Zootechnical Services (Associazione Servizi Agricoli Zootecnici [ASAZ]), an organization accredited by the Region of Piedmont for the provision of animal husbandry consultancy within the framework of the Rural Development Program (PRS) 2014/2020. The consultancy was composed of six veterinarians working chiefly in the beef fattening and veal calf industry. The farms were all clients of the ASAZ. Inclusion criteria were being a fattening operation located in Piedmont and importing beef heifers and bulls aged 8–12 months from other European countries. A 2 h seminar was conducted to improve awareness of BRD and encourage participation in the study. Participating farmers signed informed owner’s consent to authorize clinical procedures on their animals and data collection.

### 2.2. Population Sample

The estimated sample size was 146 animals based on a calculated relative risk of 4 for BRD development, a disease incidence of 18%, a 95% confidence interval, and 80% power [25,26,27]. Both relative risk and disease incidence were estimated based on previous studies, in which the lowest reported incidence averaged 4.5%, while the overall average incidence was 18% [25,26,27]. The farms were visited at least once during the study period, and at least one batch was randomly sampled and examined. Sampling was performed within 3 days after arrival at the farm. None of the animals had received treatment or vaccination prior to physical examination. Data were collected for origin, transport time, average batch weight, breed, and sex. Transport time was obtained from the official transport document. The average batch weight was obtained on arrival, when the animals were weighed by group while still on the truck. From each batch at least 15% of the animals were randomly selected. On the day of examination, all animals from the batch entered the handling corridors. Randomization was performed by selecting one animal every four or five, depending on the batch size, until at least 15% of the animals in each batch were included. On each of the selected animals a clinical examination, with a focus on respiratory signs, as well as blood sampling were performed. All animals were then vaccinated within 7 days of arrival.

### 2.3. Clinical Examination

Animals were identified by means of their ear tag, safely isolated, and submitted to clinical examination for BRD signs by an experienced veterinarian. Typical clinical signs of respiratory disease are fever (>39.5 °C), tachypnea (respiratory rate > 36 bpm), cough, nasal or ocular discharge. The farmers and the private practitioners were blinded to the results of clinical examination to avert being influenced in their treatment decisions.

### 2.4. Blood Sample Collection and Management

Blood samples were collected from the coccygeal vein with vacutainer serum and K2EDTA tubes from each animal and then transported to the laboratory of the Department of Veterinary Sciences of Turin. The samples were centrifuged at 3600× *g* for 5 min. Serum was collected and stored at −80 °C; the buffy coat was collected from the K2EDTA tubes (Becton and Dickinson, Franklin Lakes, NJ, USA), pooled, and stored at −80 °C. The samples from each batch were pooled.

### 2.5. Laboratory Analysis

Serum samples were analyzed by commercially available indirect ELISA for the detection of antibodies against bovine respiratory syncytial virus (BRSV), bovine parainfluenza-3 virus (BPIV3), bovine alphaherpesvirus 1 (BoAHV1) (Svanova Biotech, Uppsala, Sweden) and bovine viral diarrhea virus (BVDV) non-structural protein 2–3 (IDEXX Laboratories, Westbrook, ME, USA). Assays were performed according to the manufacturer’s instructions.

The buffy coat pools were sent to the laboratory of the Istituto Zooprofilattico Sperimentale del Piemonte, Liguria e Valle D’Aosta for the detection of BVDV antigens by polymerase chain reaction (PCR). Haptoglobin and reactive oxygen metabolite concentrations were determined using colorimetric methods (Diacron Labs, Grosseto, Italy; Tridelta Development Ltd., Co. Kildare, Ireland) on COBAS C501 instrumentation at the laboratory of the Istituto Zooprofilattico Sperimentale delle Venezie, Padua (ITA).

### 2.6. Pharmacological Treatment

After the initial visit, animals were examined daily by trained personnel as part of routine management and treated according to the protocols established by their veterinarians. The farmers were asked to keep a record of antibiotic treatment for BRD in the sampled calves within 60 days after their arrival. At the end of the observation period, all treatment data were extracted from the drug registers. Data are reported for the treatment of each animal, excluding prophylactic treatment. Moreover, only data for the first treatment were entered into the analysis, since they indicate initial manifestation of disease. Therapies with other drugs and/or performed for diseases not attributable to the respiratory system were not entered into the model. For the statistical analysis, we considered both 7- and 60-day periods as potential risk windows. The 60-day period was chosen based on previous reports identifying it as the period of highest risk for BRD in fattening operations [28]. The 7-day period was also considered, as the consequences of part of the observed signs were more likely to manifest within a short timeframe.

### 2.7. Statistical Analysis

Statistical analysis was performed using a freeware statistical software package (R v.4.4.0). The Shapiro–Wilk normality test was used to determine whether the data had normal distribution. Numeric data (average batch weight, transport time, reactive oxygen metabolite and haptoglobin concentration) are reported as mean and standard deviation (±SD) or as median (min, max) based on data distribution. The unit of interest for statistical analysis was the individual animals. Average batch weight and transport time are reported as categorical variables based on median and 25th and 75th percentiles and median, respectively. Average batch weight was used as a variable as the individual weight was not known, and the batches were all homogeneous among them. Categorical data (breed, sex, weight category, clinical signs, sampling season, prophylactic/metaphylactic treatment, serology) are expressed as frequency or percentage or both. Four sampling seasons were defined: spring (March, April, May); summer (June, July, August); autumn (September, October, November); and winter (December, January, February). Univariable logistic regression of all variables was performed to evaluate risk factors. All variables with a *p*-value < 0.2 were then entered in the generalized logistic models. Independent variables were: breed, sex, transport time (both as a numeric and a categorical variable, based on the median), average batch weight (with four categories according to quartile), clinical signs (tachypnea, fever, cough, nasal and ocular discharge), presence of at least one clinical sign, three or more clinical signs, positivity for antibodies for each pathogen tested, positivity for at least one, two or three of the viruses involved in BRD, reactive oxygen metabolites and haptoglobin as continuous variables. Non-continuous variables were categorized so that each level of variables included at least 10% of the data. When this was not possible, the variable was excluded from the analysis. Two separate logistic regression models were created, one for treatment within 7 days and one for treatment within 60 days. Correlation between the independent variables was tested by the chi-square test or the Wilcoxon pairwise test when numerical variables were involved. In correlations between independent variables, the one with the lowest *p*-value was retained in the final model. The most parsimonious final model was selected (lower Akaike Information Criterion [AIC]) via backward elimination, with a *p*-value of 0.05 as the removal threshold. Model fit was evaluated by Pearson’s and Hosmer–Lemeshow’s goodness-of-fit test. A value of *p* > 0.05 indicated that the data adequately fit the model. *p* was set at <0.05.

## 3. Results

### 3.1. Farm Selection

Forty-five farmers were present at the initial meeting, 25 of which met the inclusion criteria and agreed to participate in the study. Two were excluded because treatment records were unavailable. In total, 23 farms were included in the study, 78% (18/23) of which were located in the province of Cuneo and 22% (5/23) in the province of Turin.

### 3.2. Population Sample

**Figure 1:**
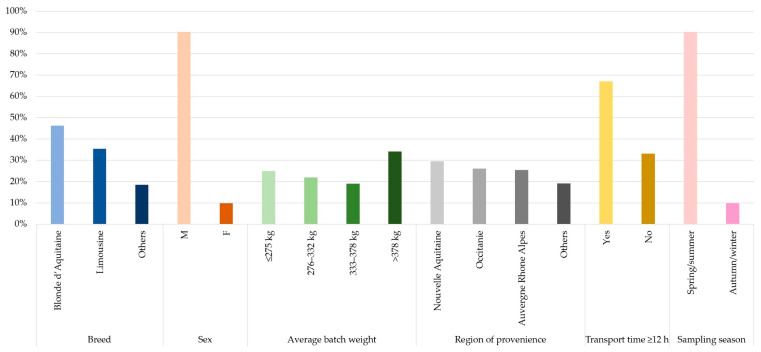
Signalment and history of the 173 beef heifers and steers included in the study. Data include breed, sex, region of provenience, transport time ≥ 12 h, average batch weight categories (divided by quartiles), sampling season. M: male; F: female. Other Breed category: Charolaise (n = 15) and mixed breed (n = 17) from Bourgogne-Franche-Comté (n = 14), Haute-de-France (n = 2), Bretagne (n = 3), Centre-Val de la Loire (n = 7), Grand Est (n = 6), and Pays de la Loire (n = 1). Data are reported as percentages.

A total of 26 animal batches were evaluated. Only one batch was examined on 20 farms and two batches on the remaining three farms. Of the total of 760 calves transported and introduced on the farms, 173 (22.8%) were entered in the study. The median percentage of animal sampled per batch was 22% (range, 16–46). Sampling was carried out in spring (59%) and summer (26%); no farms were sampled in winter. The two most numerous breeds were Blonde d’Aquitaine (80/173, 46.2%) and Limousine (61/173, 35.3%), followed by Charolaise (15/173, 8.7%) and mixed breed (17/173, 9.8%). These latter were collectively classified as “Others” (32/173, 18.5%). The median batch weight was 332 kg (range, 195–470). Most of the animals originated from four French regions: Nouvelle-Aquitanie (51/173, 29.5%), Occitanie (45/173, 26.0%), Auvergne-Rhône-Alpes (44/173, 25.4%), while the remaining (33/173, 19.1%) were from: Bourgogne-Franche-Comté (n = 14), Haute-de-France (n = 2), Bretagne (n = 3), Centre-Val de la Loire (n = 7), Grand Est (n = 6), and Pays de la Loire (n = 1). These animals were collectively classified as originating from Other Regions (33/173, 19.1%). The median transport time was 12 h (range, 4.5–18). None of the batches stopped in a lairage during transport. Figure 1 presents the data for animal history and signalment. All farms vaccinated within 7 days of arrival against three viral agents (BRSV, BoAHV-1, BVDV); 21 also vaccinated against *Mannheimia haemolytica* serotype A1), and 21 administered parasiticide treatment within 7 days of arrival.

### 3.3. Clinical Examination

**Figure 2:**
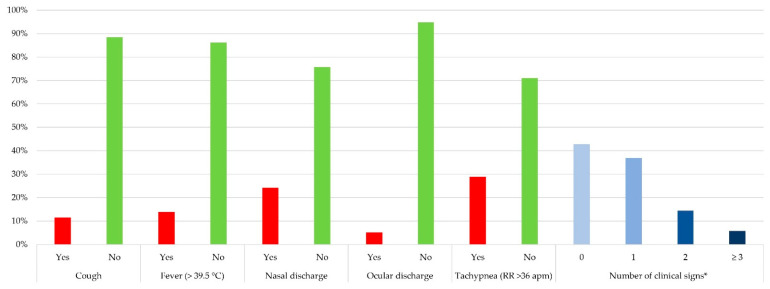
Data about clinical examination of the 173 beef heifers and steers included in the study. * Among the 5 collected during the study. Data are reported as percentage.

Figure 2 presents the frequency of clinical signs. The most frequent clinical sign detected on arrival was tachypnea (50/173, 28.9%), followed by nasal discharge (42/173, 24.3%). At least one clinical sign was noted in 57.2% (99/173) of the animals, most (64/99, 64.6%) of which showed only one clinical sign.

### 3.4. Laboratory Analysis

**Figure 3:**
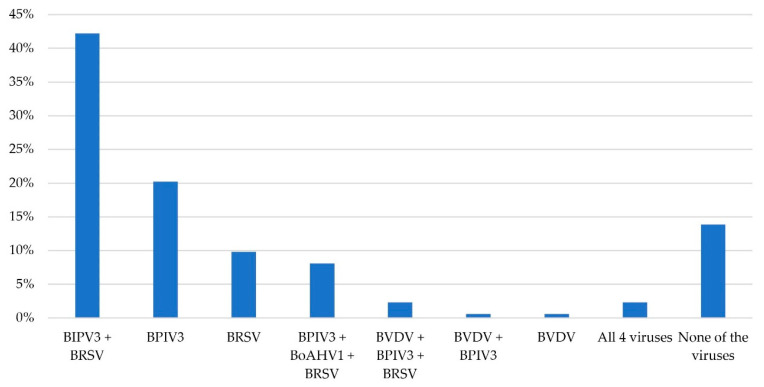
Combination of seropositivity for the 173 beef heifers and steers included in the study. BPIV3: bovine parainfluenza-3 virus; BRSV: bovine respiratory syncytial virus; BoAHV1: bovine alphaherpesvirus type 1; BVDV: bovine viral diarrhea virus. Data are reported as percentages.

Serological analysis revealed that most animals tested positive for PI-3 (131/173, 75.7%) and BRSV (112/173, 64.7%), while far fewer were found to have antibodies against BoAHV-1 and BVDV (18/173, 10.4% and 10/173, 5.8%, respectively). Four animals tested positive for all viruses (4/173, 2.3%), 18 were positive for three (18/173, 10.4%), 74 were positive for two (74/173, 42.8%), 53 were positive for only one virus (53/173, 30.6%), and 24 tested negatives for all viruses (24/173, 13.9%). Combinations of seropositivity are reported in Figure 3. The pooled samples were negative for BVDV PCR.

### 3.5. Pharmacological Treatment

The animals were vaccinated within 7 days of arrival on the farm. All farms vaccinated against at least three viruses (BRSV, BoAHV-1, BVDV), while 21 farms also vaccinated against *Mannheimia haemolytica* serotype A1, and 21 administered parasiticide treatment within 7 days of arrival. During the first 60 days of fattening operations, 34 animals (34/173, 19.7%) were treated for BRD, 13 of which (13/34, 38.2%; 13/173, 7.5%) were treated within the first 7 days. The median time interval between arrival and BRD treatment was 13 days (range, 0–31). Three of the batches received metaphylactic/prophylactic treatment after arrival, with 18 animals receiving treatment as a group (18/173, 10.4%). The average batch weight at arrival of these animals was ≤275 kg. No animals died during the observation period.

### 3.6. Reactive Oxygen Metabolites and Haptoglobin

Table 1 presents the value of levels of reactive oxygen metabolites and haptoglobin in animals treated within 7 and 60 days.

### 3.7. Univariate and Multivariate Analysis

Table 2 and Table 3 present the variables that were included in the multivariate models of risk factors for developing BRD. Breed, weight, positivity for at least three viruses, province of origin, and transport time (categorized based on median) were all correlated (*p* < 0.05). There was a statistically significant difference in reactive oxygen metabolite concentration between Blonde d’Aquitaine (median, range, 84, 40–192 U/CARR) and Limousine (median, range, 100, 58–228 U/CARR). There were differences in risk between animals imported from Occitanie (median, range, 78, 40–150 U/CARR) and those from Nouvelle-Aquitaine (median, range, 92, 58–192 U/CARR) and “Other Provinces” (median, range, 109, 40–208 U/CARR). The reactive oxygen metabolite level was lower in the animals that had a transport time ≥12 h (median, range, 84.5, 40–192 U/CARR) than those transported for <12 h (median, range, 108, 40–228 U/CARR) (*p* < 0.05). The variables retained in the final models were average weight on arrival and reactive oxygen metabolite level for treatment within 7 days and average weight on arrival, reactive oxygen metabolite and transport time for treatment within 60 days of arrival (Table 4). Treatment within 7 days of arrival correlated with weight > 378 kg and reactive oxygen metabolite level. Treatment within 60 days correlated with weight between 276 kg and 332 kg and weight > 378 kg. Treatment within 60 days also correlated with transport time > 12 h.

## 4. Discussion

The rate of participation in the project indicates that BRD in imported cattle remains an issue for both veterinarians and farmers. Indeed, nearly 80% of the farmers invited agreed to participate. In line with previous findings, the prevalence of BRD based on treatment records was 19.2% [11,27,29]. Moreover, about half of the animals received treatment in the first 7 days on arrival at the farm, and the remaining animals completed treatment within the first 31 days on feedlot, consistent with the common description of BRD incidence, which usually develops in the first days after arrival [8,11,15,28].

Our findings show that weight on arrival, transport time > 12 h, and reactive oxygen metabolite level are predisposing factors for BRD treatment in the first days of the fattening period. Weight has been reported as a potential factor for BRD, with lighter-weight animals at greater risk of developing signs of BRD [4,8,9,30]. Our data contrast with these latter; however, the models indicated a higher risk for heavier animals of being treated in the first days after arrival. Weight categories may differ between studies, but those reporting a weight effect concur that lighter weight animals are at higher risk of developing BRD. This is logical, given that lighter animals are usually younger ones, with a less mature immune system or retarded growth due to their clinical history [9]. A possible explanation for our results is that some of the lighter animals (n = 18) received group treatment a few days after their arrival, which arguably may have protected them against further infection. Moreover, the fact that they were treated as a group makes it difficult to determine whether they really developed BRD or were treated preventatively, since others in the same group were found sick. If we consider only individual treatment and if some of these calves became sick, they were not entered in the analysis, which may have created a possible bias. Indeed, since we were unable to determine whether they were treated because they were truly sick or because they were part of a risk group, their inclusion in the analysis would likewise have created a bias in the opposite direction.

Regarding administration of treatment within 7 days, two categories of the weight variable (276–332 kg; 333–378 kg) had an OR of 0 and infinite 95% CI. This may have been due to complete separation of the data, meaning that one of the cells of a 4 × 4 table has a value of 0 [31], a problem usually associated with a sample size that is too small [31]. Furthermore, in the present study, the prevalence of treatment for BRD within 7 days of arrival was lower than the prevalence estimated for the sample size calculation; a larger sample would have been needed in order to evaluate the effect of weight.

The steers and heifers transported for longer had a higher risk of being treated for BRD. Previous studies have reported an association between transport time and increased risk of BRD in beef cattle and infection of the upper respiratory tract with potentially pathogenic microorganisms [32,33,34]. Upper respiratory tract infection can induce behavioural and biochemical alterations in cows and beef cattle and reduce immune response, thus masking the presentation of clinical signs upon arrival [14,35,36]. The fact that this variable was significant only for treatment within 60 days was perhaps due to too small a sample size for treatment administered within 7 days.

Other possible predisposing factors for BRD development are season of arrival and sex. A higher incidence of BRD in autumn or winter has been previously reported [9,37,38,39]. Notably, autumn and winter are characterized by rapid changes in temperature and adverse weather conditions, which place additional stress on cattle, especially in correlation with other stressful events (transport, commingling) [40,41]. Unfortunately, data for the fall/winter seasons were too few to be included in the analysis.

Male calves are reported to be more susceptible to developing BRD [9,28,42,43], which can be correlated with the fact that many calves arrive at the feedlot as bulls and are then castrated [44]. Since castration is a painful and stressful event, it is thought to increase susceptibility to the development of BRD [44]. As too few females were included in the study, this variable was not entered in the analyses.

Breed was identified as a possible predisposing factor only by the univariate analysis. Previous reports, which included many different breeds, reported an association between breed and the development of BRD [10,45] and an increase in antimicrobial use in batches of Limousine or Blonde d’Aquitaine [46], both of which were numerous in our study. We noted that breed was correlated with weight, which might explain why it was not retained in the final model. Weight was probably a stronger variable than breed for explaining the possible development of BRD. While we found no correlation between region of origin and treatment, an earlier French study reported a geographical distribution of BRD incidence in certain French departments [46]. Our analysis was based on region of origin, so as to avoid excessively small groups for analysis. The distribution of BRD incidence in the main animal-exporting regions was heterogeneous, based on a report by Gay and Barnouin [46]. However, since a production-type distribution was not given, we could not obtain information about the specific category of interest.

The presence of clinical signs on arrival was not correlated with subsequent treatment for BRD. Most of the animals had one clinical sign or none; the most common was tachypnea, which is not specific for respiratory disease and respiratory rate may increase owing to a stressful event [12,47]. Elevated body temperature cannot be considered a specific sign of BRD either, since it usually increases during transport and in relation to animal temperament [48]. Moreover, there is no consensus on the cut-off body temperature for a diagnosis of BRD and it is not usually used for defining clinical BRD [49]. Given the effect of transport on body temperature, the cut-off temperature we used may have led to a misclassification of healthy and BRD-affected calves. Furthermore, clinical examination has low accuracy and is operator-dependent [49,50], which may explain why we found no correlation between clinical signs on arrival, as determined by us, and treatment by the farmers or the veterinarians.

The high seroprevalence of BRSV and BPIV3 antibodies we observed is also described by Hay et al. [17] in feedlot cattle. The seroprevalence reported by Hay et al. [17] and by Durham et al. [51] for BVDV and BoAHV-1 was higher than ours. This was probably due to French eradication plans to reduce the prevalence of the two diseases. The nation-wide, mandatory French BoAHV1 eradication plan, started in 2006, calls for the vaccination of herds with positive animals, but not herds found to be BoAHV-1-free [52,53]. The BVDV eradication plan is mandatory in certain departments and voluntary but strictly recommended by the Groupements de Défense Sanitaire in the rest of France. The aim is to identify and eliminate immunotolerant, persistently infected calves and the serology for adult cattle [54]. Contrary to previous studies that found a correlation between seropositivity and a reduced risk for BRD, we noted no association between the detection of viral antibodies and BRD treatment [50,52,55,56]. Given that all the farms operated a vaccination protocol that included BoAHV1, BRSV, and BVDV, and that 17 farms vaccinated for BPIV3 as well, a correlation between serology results and treatment in the first days of arrival is difficult to determine. However, since the farmers had little information about animal health management before transport, the results hold interest for the difference in seroprevalence within and between the batches. The inclusion of viral pathogens only was based on their role as potential predisposing factors for bacterial infection [17]. However, to fully understand the impact of prior infections on subsequent BRD development, further studies including serology against bacterial pathogens are needed.

Haptoglobin level on arrival was not related to BRD treatment in the first days after arrival. An acute phase protein, haptoglobin non-specifically increases in response to inflammation, infection or stress. It is known to increase during BRD, although mainly correlated with greater tissue damage, chronic disease, and bacterial infection. Nonetheless, its practical application in cattle remains debated [57,58,59]. Reactive oxygen metabolite levels were associated with BRD treatment within 7 or 60 days in the present study. In particular, the results of the present study showed that animals with lower concentrations of ROMs at arrival were at higher risk of developing BRD. Reactive oxygen metabolites are natural byproducts of oxygen metabolism and are involved in physiological processes and modulation of inflammatory response [24]. Oxidative stress occurs when reactive oxygen metabolite production exceeds antioxidant availability [24,60]. To our best knowledge, few studies to date have investigated reactive oxygen metabolite levels in beef calves. However, previous studies showed that animal`s transport led to an oxidative stress demonstrated by the rise in oxidative stress biomarkers, which can predispose for and be correlated with the development of BRD [22].

Elevated reactive oxygen metabolite levels in human patients can be harmful as can too low a concentration, reflecting reduced antimicrobial defence [24]. In detail, mitochondrial respiration is not the only source of reactive oxygen metabolites, as they can also be synthesized by NADPH oxidase enzymes (NOX) [60]. In human patients, NOX activity is influenced by genetic variability and so, too, the amount of reactive oxygen metabolites produced [24]. One of the possible explanations for our results and their contrast with previous studies is that a similar genetic component is also present in cattle. Animals with lower reactive oxygen metabolite production may be more susceptible to developing BRD. However, given the limited number of animals in the present work, no firm conclusions can be drawn. Further studies are needed to clarify the role of ROMs as potential predictors of BRD.

Identifying animals at higher risk of developing BRD upon arrival at the fattening operation can have important implications for the industry. It can provide valuable information to farmers and veterinarians, supporting improved management of high-risk animals.

## 5. Conclusions

Our data indicate that transport time and average weight at arrival of animals imported from France were the two major factors associated with BRD treatment in the first days after arrival at local fattening operations. Furthermore, while haptoglobin level was not found to be a good predictor for BRD treatment, reactive oxygen metabolite levels may be useful for predicting the potential development of BRD. Further studies are needed to draw conclusions, however.

## Figures and Tables

**Table 1:** Values (range) of haptoglobin (HP) and reactive oxygen metabolites (ROM) are reported as median (min, max), based on treatment within 7 or 60 days of arrival.

Parameter	Category	ROM (U/CARR)	HP (mg/dL)
Treatment within 7 days	Yes	62 (40, 192)	33 (15, 78)
	No	92 (40, 228)	31 (11, 280)
Treatment within 60 days	Yes	70 (40, 192)	32.5 (11, 280)
	No	95 (40, 228)	30 (11, 150)

**Table 2:** Univariate analysis of variables in the multivariable model (*p* ≤ 0.2) assessing factors predisposing for treatment within 7 days of arrival for 173 beef calves imported in northwestern Italy fattening operation. OR: Odds Ratio; CI: Confidence interval; ROMs: reactive oxygen metabolites. * Other Breed category: Charolaise and mixed breed. ** Other Province category: Bourgogne-Franche-Comté, Haute-de-France, Bretagne, Centre-Val de la Loire, Grand Est, and Pays de la Loire.

Variable	Category	OR	95% CI	*p* Value
Breed	Blonde d’Aquitaine	Referent	-	-
	Limousine	0.21	0.01–1.25	0.148
	Others *	2.85	0.82–9.87	0.092
Average weight on arrival	≤275 kg	Referent	-	-
	276–332 kg	0	NA–∞	0.995
	333–378 kg	0	NA–∞	0.996
	>378 kg	10.72	1.98–199.49	0.026
Positivity to at least three viruses	No	Referent	-	-
	Yes	3.51	0.8–12.02	0.054
Province of origin	Auvergne-Rhone-Alpes	Referent	-	-
	Nouvelle Aquitaine	0.42	0.02–4.53	0.485
	Occitanie	3.23	0.7–22.96	0.166
	Others **	2.9	0.53–21.9	0.237
ROMs	-	0.97	0.94–0.99	0.01

**Table 3:** Univariate analysis of variables in the multivariable model (*p* ≤ 0.2) assessing factors predisposing for treatment within 60 days of arrival for 173 beef calves imported in northwestern Italy fattening operation. OR: Odds Ratio; CI: Confidence interval; ROMs: reactive oxygen metabolites. * Other Breed category: Charolaise and mixed breed. ** Other Province category: Bourgogne-Franche-Comté, Haute-de-France, Bretagne, Centre-Val de la Loire, Grand Est, and Pays de la Loire.

Variable	Category	OR	95% CI	*p* Value
Breed	Blonde d’Aquitaine	Referent	-	-
	Limousine	0.29	0.1–0.72	0.012
	Others *	0.61	0.2–1.6	0.337
Average weight on arrival	≤275 kg	Referent	-	-
	276–332 kg	0.28	0.06–1.02	0.072
	333–378 kg	0.33	0.07–1.2	0.116
	>378 kg	1.45	0.6–3.66	0.419
Transportation time ≥ 12 h	No	Referent	-	-
	Yes	3.47	1.36–10.68	0.016
Province of origin	Auvergne-Rhone-Alpes	Referent	-	-
	Nouvelle Aquitaine	0.84	0.26–2.67	0.765
	Occitanie	2.64	0.98–7.70	0.061
	Others **	0.94	0.26–3.27	0.928
ROMs	-	0.98	0.96–0.99	0.002

**Table 4:** Multivariate analysis of the models assessing factors predisposing for treatment within 7 and 60 days of arrival for 173 beef calves imported in northwestern Italy fattening operation. OR: Odds Ratio; CI: Confidence interval; ROMs: reactive oxygen metabolites; AIC: Akaike’s information criterion.

		Treatment Within 7 Days			Treatment Within 60 Days		
Variable	Category	OR	95% CI	*p* Value	OR	95% CI	*p*-Value
Average weight on arrival	≤275 kg	Referent	-	-	Referent	-	-
	276–332 kg	0	NA–∞	0.995	0.22	0.05–0.83	0.036
	333–378 kg	0	NA–∞	0.996	0.43	0.09–1.67	0.252
	>378 kg	11.54	2.07–216.9	0.023	3.09	1.09–9.4	0.039
Transportation time ≥ 12 h	No	-	-	-	Referent	-	-
	Yes	-	-	-	6.65	2.13–24.65	0.002
ROMs	-	0.98	0.96–0.99	0.023	0.99	0.97–1	0.056
AIC	-	71.291			148.62		
R^2^	-	0.236			0.213		

## Data Availability

Data available from corresponding author upon reasonable request due to privacy restriction.

## Abbreviations

The following abbreviations are used in this manuscript:

BRD	Bovine respiratory disease
BRSV	Bovine respiratory syncytial virus
BPIV3	Parainfluenza-3 virus
BoAHV1	Bovine herpesvirus 1
BVDV	Bovine viral diarrhea virus
PCR	Polymerase chain reaction
HP	Haptoglobin
ROM	Reactive oxygen metabolites
